# Association between central corneal thickness and systemic lupus erythematosus: a cross-sectional study protocol

**DOI:** 10.3389/fmed.2024.1483930

**Published:** 2024-10-15

**Authors:** Juan David Saldaña-Garrido, Mario Cantó-Cerdán, Vicente Francisco Gil-Guillén, María Luisa Alfaro-Beltrá, Francisca Sivera

**Affiliations:** ^1^Department of Ophthalmology, General University Hospital of Elda, Elda, Spain; ^2^Department of Clinical Medicine, School of Medicine, Miguel Hernández de Elche University, San Juan de Alicante, Spain; ^3^Vissum Miranza, Alicante, Spain; ^4^Department of Investigations, General University Hospital of Elda, Elda, Spain; ^5^Department of Rheumatology, General University Hospital of Elda, Elda, Spain

**Keywords:** systemic lupus erythematosus, central corneal thickness, pachymetry, optical coherence tomography, glaucoma, hydroxychloroquine

## Abstract

**Introduction:**

Systemic lupus erythematosus (SLE) is a chronic autoimmune disease affecting multiple systems and classified under connective tissue disorders. Ocular involvement occurs in up to 30% of SLE cases, with the cornea being particularly susceptible to thinning due to immune-complex deposits and its predominantly type I collagen composition. This corneal thinning is clinically significant in glaucoma, where patients with reduced central corneal thickness (CCT) may have up to a threefold increased risk of developing glaucoma, as well as in refractive surgery. However, existing studies on CCT in SLE are limited and marked by substantial heterogeneity in methodology, technology, criteria, and participant numbers, resulting in conflicting findings. Based in our hypothesis that SLE-related corneal lysis may result in decreased CCT, this study aims to determine and compare the mean CCT values between SLE patients and healthy controls to obtain a more precise understanding of the potential relationship.

**Methods and analysis:**

A cross-sectional observational study will be conducted, enrolling SLE patients and age-and sex-matched healthy controls recruited from ophthalmology consultations. Exclusion criteria will be applied to rule out other corneal thinning risk factors. A pilot study estimated a minimum sample size of 34 participants per group. CCT measurements will be obtained using Zeiss HD Cirrus 5,000 optical coherence tomography (OCT) on a randomly selected eye, following concordance analysis using the Kappa index. Statistical analysis will include descriptive, bivariate, and multivariate methods. The study protocol was approved by the ethics committee.

**Discussion:**

The cornea’s vulnerability to thinning and lysis in SLE, which impacts CCT, is crucial for the accurate assessment of glaucoma, the leading cause of irreversible blindness worldwide and the second leading cause in Europe. Given that patients with reduced CCT are at a significantly higher risk of developing glaucoma, further research is necessary to understand the association between SLE and CCT. Our study aims to enhance methodological rigor compared to prior research by determining an appropriate sample size and exclusively enrolling SLE patients to increase participant homogeneity. If a significant difference in CCT between groups and an association between CCT and SLE are found, a prospective study will be considered.

## Introduction

Systemic lupus erythematosus (SLE) is a chronic autoimmune multisystemic disease of unknown etiology, classified under “connective tissue diseases.” It is relatively rare, with a prevalence of 39 per 100,000 in Europe (1), and is more common among young women and individuals of Black, Asian, and Hispanic descent (2). The European League against Rheumatism (EULAR)/American College of Rheumatology (ACR) developed classification criteria for SLE, with a sensitivity and specificity of 96.1 and 93.4%, respectively (3). However, not all patients meet these criteria, complicating and delaying diagnosis.

Regarding SLE manifestations, most patients experience constitutional syndrome (fever, weight loss, and asthenia) at some point during the disease (1). The musculoskeletal system, skin and kidney are also commonly affected (4). However, the eye can also be involved, with ocular manifestations in up to 30% of patients (1, 5).

Most ocular alterations are not considered in the overall assessment of SLE, as recognized by the British Isles Lupus Assessment Group (BILAG) (1). However, these disorders often occur in the context of systemic disease activity, with many being asymptomatic (1, 6), delaying specific treatment and worsening visual prognosis. This suggests that ophthalmologic evaluation could be key for diagnosing and especially monitoring SLE activity.

These ocular manifestations vary widely and can affect nearly any ocular structure. Common changes include secondary dry eye syndrome—due to secondary Sjögren’s syndrome (1, 5)—and bilateral small vessel retinal vasculitis (1, 4, 5, 7). Other manifestations include recurrent corneal erosions, stromal corneal infiltration, corneal opacity, peripheral ulcerative keratitis, corneal edema, interstitial keratitis (4), choroidal effusion, optic neuropathy (1), as well as orbital or eyelid inflammation, and various retinal vascular alterations such as vascular occlusions related to associated antiphospholipid syndrome (1).

The cornea, primarily composed of type I connective tissue (4, 5), is particularly vulnerable to thinning and lysis phenomena in SLE (4, 8) due to inflammation triggered by autoantibodies (specifically anti-double-stranded DNA antibodies—anti-dsDNA—and anti-Smith antibodies—anti-Sm) and immune complexes deposited in its basement membrane (4). Consequently, various research efforts have aimed to elucidate the relationship between SLE and the central corneal thickness (CCT). These investigations were conducted by Zang et al. (9), Çağlayan et al. (10), Yazici et al. (4), Mahendradas et al. (8), Eissa et al. (5), Kaya et al. (11) and Mahmoud et al. (6).

These previous studies examining CCT in SLE patients report conflicting results. Çağlayan et al. (10) and Zhang et al. (9) reported thicker CCT in SLE compared to controls. Conversely, Yazici et al. (4), Mahendradas et al. (8), Eissa et al. (5), Kaya et al. (11) and Mahmoud et al. (6), reported a reduced CCT in SLE compared to controls (4–6, 8, 11).

Additionally, these studies present certain limitations that hinder the extraction of definitive conclusions. The study by Zhang et al. (9) does not directly measure CCT, but rather evaluates corneal hysteresis and assumes an association with CCT based on previous studies. Regarding sample size, several investigations do not provide a sample size calculation and exhibit highly variable sizes. The studies by Mahendradas et al. (8) and Oğurel et al. (12) are notable for their particularly small samples 7 and 4 patients with SLE, respectively, sometimes only performing a descriptive statistical analysis of the SLE patients, as in the case of Mahendradas et al. (8), or analyzing both eyes, as in the case of Mahmoud et al. (6).

Furthermore, there are differences in the inclusion of patients with active or inactive SLE between the studies. While the studies by Yazici et al. (4), Zhang et al. (6), and Mahmoud et al. (9) do not mention the activity status of SLE, others, such as Mahendradas et al. (8), include patients with both active and inactive SLE. The study by Eissa et al. (5) includes only patients with active SLE, whereas the studies by Kaya et al. (10) and Çağlayan et al. (11) focus exclusively on patients with inactive SLE.

Measurement of CCT, a biomechanical property of the cornea, is essential for interpreting intraocular pressure (IOP) measurements (13) and it is considered one of the main sources of error in applanation tonometry (14). A thicker cornea (greater CCT) leads to artificially increased IOP measurements, whereas a thinner cornea (lower CCT) results in an underestimation of IOP (13). Therefore, evaluating CCT is crucial for diagnosing significant conditions such as glaucoma or keratoconus, as well as for assessing suitability for corneal refractive surgery techniques such as Laser Assisted *in Situ* Keratomileusis (LASIK) or photorefractive keratectomy (PRK) (14–16).

Glaucoma is the leading cause of irreversible blindness globally (17) and the second leading cause in Europe (18), with an estimated 111.8 million people projected to have glaucoma by 2040 (17). Glaucoma is typically asymptomatic until very advanced stages, with up to 50% of cases going undiagnosed (18). Despite its unknown etiology, elevated IOP is the primary risk factor for developing glaucoma (15, 18), making IOP and CCT evaluation essential in clinical practice. In SLE, this risk may be exacerbated by the disease’s distinctive vascular characteristics, including retrobulbar artery narrowing and increased sensitivity to vasoconstriction (7).

Additionally, CCT measurements are crucial for predicting potential outcomes or complications of refractive surgery in SLE patients, who predominantly fall within the 30–50 age range and are therefore in their productive years (4). Misdiagnosis or inappropriate treatment strategies could significantly impact their quality of life and increase healthcare costs.

Apart from disease manifestations, it is important to consider the ocular toxicity and side effects of medications used to treat SLE. Commonly prescribed drugs include corticosteroids and hydroxychloroquine (HCQ). Corticosteroids can induce cortical or posterior subcapsular cataracts and secondary glaucoma (1). HCQ, meanwhile, can affect the cornea (19), leading to verticillate keratopathy (1), changes in endothelial cell density and may result in CCT thickening (10, 12). HCQ can also have an impact on the ciliary body and retinal pigment epithelium (RPE), causing maculopathy (1). These side effects necessitate regular ophthalmologic monitoring as they can pose a significant threat to vision (1).

Given the cornea’s susceptibility to thinning and lysis phenomena in SLE (4, 8), and considering the limited number of studies on CCT and SLE relationship and their conflicting conclusions, further investigations to enhance our understanding of this association is crucial. Therefore, based on the hypothesis that CCT is reduced in SLE patients compared to healthy individuals, we are conducting this cross-sectional study with the main objective of determining whether there are differences in mean CCT between SLE patients and healthy controls.

## Methods and analysis

### Aims and objectives

The primary aim of this study is to determine and compare the mean CCT values between SLE patients and age-and sex-matched healthy controls using Zeiss HD Cirrus 5,000 optical coherence tomography (OCT). Additionally, the study aims to enhance methodological rigor relative to prior research by determining sample size and exclusively enrolling SLE patients to increase participant homogeneity.

Other secondary objectives are:

- To determine the agreement of CCT between the right eye and left eye in both the SLE group and the healthy control group.
- To describe the proportion of patients with decreased and normal CCT in both the SLE group and the healthy control group.
- To compare glaucoma-related variables – IOP, retinal nerve fiber layer (RNFL) thickness, and visual field (VF)—between the SLE group and the healthy control group.
- To identify the number of new glaucoma diagnoses in SLE group.
- To establish the association between patient and disease characteristics and CCT in patients with SLE.

### Study design

Based on the hypothesis and the main objective, we are conducting a cross-sectional observational study at the Department of Ophthalmology at General University Hospital of Elda, in collaboration with the University of Miguel Hernández of Elche, Spain, where participants will be part of one of two groups:

- Group 1: patients with SLE diagnosis, without ocular or systemic pathology affecting CCT.
- Group 2: patients without SLE diagnosis, without ocular or systemic pathology affecting CCT.

## Study subjects

### Study population and sampling method

Catchment population includes all patients served by the Health Department of Elda (~200,000 patients). Study participants included both healthy control subjects and patients diagnosed with SLE, who meet all inclusion criteria, but none of the exclusion criteria. Patients will be recruited consecutively.

### Sample size calculation

Given the limited available literature, with few studies, heterogeneous sample sizes and conflicting results, there is insufficient evidence to estimate the necessary sample size. Therefore, a pilot project was conducted with 20 patients (10 patients with SLE and 10 healthy patients) where the mean CCT value was 525 ± 25 μm for the group of patients with SLE and 510 ± 24 μm for the group of healthy patients. Establishing a bilateral hypothesis with a type 1 error or *α* of 5% and a type 2 error or *β* of 20% (equivalent to a power of 80%), the resulting effect size was 0.61. This calculation indicated a requirement of a minimum of 34 participants per group.

### Inclusion criteria

- Patients 18 years of age or older.
- Patients diagnosed with and under follow-up for SLE by the Rheumatology Department of the Health Department of Elda, and not exhibiting disease activity according to the treating rheumatologist.
- Patients without SLE and without ocular or systemic pathology affecting the CCT (as specified in the exclusion criteria), from the Health Department of Elda.

### Exclusion criteria

- Pregnancy or lactation.
- Patients of African descent.
- Previous ocular disease that may affect the CCT (keratoconus, corneal edema, uveitis) or previous diagnosis of glaucoma.
- Patients with systemic diseases that may affect the CCT (diabetes mellitus, chronic obstructive pulmonary disease—COPD).
- Severe astigmatism (> 3 diopters) or severe myopia [axial length (AL) > 26 mm or sphere ≥ − 6 diopters].
- Patients undergoing topical ocular treatment, except for artificial tears or with a history of ophthalmic surgery and regular contact lens users.
- Patients treated with topical or inhaled corticosteroids in the last 3 months.
- Patients receiving periocular corticosteroids or systemic prednisone at doses ≥7.5 mg/day in the last 6 months.

## Study variables

### Qualitative dichotomous variables

- SLE (Yes/No)
- Hydroxychloroquine (Yes/No)
- Sex (Male/Female)
- Schirmer Test Type 2 (Normal/Abnormal): Considered abnormal when the test shows <10 mm wetting after 5 min.
- VF 24–2 (Glaucomatous/Non-glaucomatous): A glaucomatous VF will be considered when typical glaucoma defects are present: defects not respecting the vertical line but respecting the horizontal line, Rönne’s nasal step, Bjerrum’s arcuate defects, and centrocecal defects.
- Raynaud’s Phenomenon (Yes/No)
- Constitutional Syndrome (fever, fatigue, and weight loss)
  - Previous (Yes/No)
  - Current (Yes/No)
- Mucocutaneous Involvement (malar rash, oral or nasal ulcers, photosensitivity)
  - Previous (Yes/No)
  - Current (Yes/No)
- Musculoskeletal Involvement (arthritis, arthralgia)
  - Previous (Yes/No)
  - Current (Yes/No)
- Renal Involvement (glomerulonephritis)
  - Previous (Yes/No)
  - Current (Yes/No)
- Cardiac Involvement (pericarditis)
  - Previous (Yes/No)
  - Current (Yes/No)
- Gastrointestinal Involvement (esophagitis, peritonitis, lupus hepatitis, pancreatitis)
  - Previous (Yes/No)
  - Current (Yes/No)
- Pulmonary Involvement (pleuritis, pneumonitis)
  - Previous (Yes/No)
  - Current (Yes/No)
- Neuropsychiatric Involvement (delirium, psychosis, depression, anxiety, epilepsy, motor disorder)
  - Previous (Yes/No)
  - Current (Yes/No)
- Hematologic Involvement (anemia, leukopenia, thrombocytopenia)
  - Previous (Yes/No)
  - Current (Yes/No)
- Ocular Involvement (dry keratoconjunctivitis, retinal vasculitis, scleritis/episcleritis)
  - Previous (Yes/No)
  - Current (Yes/No)
- Current use of Methotrexate (Yes/No)
- Current use of Mycophenolate (Yes/No)
- Current use of Azathioprine (Yes/No)
- Current use of Belimumab (Yes/No)
- Current use of Leflunomide (Yes/No)
- Current use of Tacrolimus (Yes/No)

### Qualitative ordinal variables

- CCT: This is the main variable. It will be coded according to the following classification:
  - Decreased: < 510 μm. CCT is classified as decreased when measurements fall below the threshold defined as normal by the European Glaucoma Society (EGS), specifically values less than 510 μm.
  - Normal: > 510 μm. In categorizing CCT as “normal,” all participants with CCT values falling within the normal range (510–570 μm) or higher (> 570 μm) are grouped together. CCT is considered normal or increased if measurements are equal to or greater than the thresholds established by the EGS, specifically starting from 510 μm.
- Color Code for Average RNFL Thickness of the Optic Nerve, measured by OCT:
  - Normal: Green
  - Abnormal: Yellow or Red
- Antinuclear Antibody Pattern
  - Nuclear Dots
  - Nucleolar
  - Speckled
  - Homogeneous
  - Cytoplasmic
  - Centromere

### Quantitative discrete variables

- CCT (μm). Considered as the dependent variable, it is the main variable.
- IOP measured by Goldman applanation tonometry (GAT) (mmHg)
- Average RNFL Thickness of the Optic Nerve, measured by OCT (μm)
- Duration of SLE Disease (years)
- Time since last inflammatory flare-up (months)
- Age (years)
- Antinuclear Antibody Titers (fraction)
- Duration of hydroxychloroquine use (months)

### Quantitative continuous variables

- Sphere (diopters)
- Cylinder (diopters)
- Spherical Equivalent (SE) (diopters): This simplifies and effectively represents the behavior of the optical system using an ideal spherical lens that represents the circle of least confusion where a clear image is produced in the patient, facilitating analysis. It is the result of the sum of the spherical refractive error and half of the cylindrical refractive error of the patient.
- AL (mm)
- Anti-dsDNA Antibodies (IU/mL). For statistical analysis, participants with values <9.8 IU/mL were assigned a value of 0, as values below 9.8 IU/mL are clinically considered “undetectable,” allowing for clearer statistical analysis.
- Anti-Smith Antibodies (U/mL). For statistical analysis, participants with valued <3.3 U/mL were assigned a value of 0, as values below 3.3 U/mL are clinically considered “undetectable,” allowing for clearer statistical analysis.
- Anti-Ro Antibodies (U/mL). For statistical analysis, participants with values <2.3 U/mL were assigned a value of 0, as values below 2.3 U/mL are clinically considered “undetectable,” allowing for clearer statistical analysis.
- Anti-La Antibodies (U/mL). For statistical analysis, participants with values <3.3 U/mL were assigned a value of 0, as values below 3.3 U/mL are clinically considered “undetectable,” allowing for clearer statistical analysis.
- Keratometry 1 (K1) -flat- (diopters): Measures the anterior curvature in the central 3 mm of the cornea in its flattest meridian. Normal value is 43–44 diopters.
- Keratometry 2 (K2) -steep- (diopters): Measures the anterior curvature in the central 3 mm of the cornea in its steepest meridian. Normal value is 43–44 diopters.
- Average Keratometry (KM) (diopters): Measures the average anterior curvature in the central 3 mm of the cornea. Normal value is 43–44 diopters.

## Data analysis

### Statistical analysis

The statistical analysis will be performed using the Statistical Package for the Social Sciences (SPSS) version 26.0 developed by IBM Corp. (Armonk, NY) for Windows. The study will be conducted, first considering the whole sample, and additionally, a sub-analysis will be carried out considering only patients with SLE.

To determine the concordance between the right eye and the left eye to decide whether both eyes or only one eye per participant will be analyzed, a concordance analysis using the Kappa index will be performed.

### Descriptive analysis based on the type of variable

Proportions will be calculated for qualitative variables, while mean and standard deviation will be computed for quantitative variables. Additionally, 95% confidence intervals will be determined for the most pertinent variables.

### Bivariate analysis

The normality of the variables will be determined using the Kolmogorov–Smirnov test. Parametric tests will be employed if the dependent variable conforms to a normal distribution: when comparing two qualitative variables (two proportions), the Chi-Square test will be used. When comparing a qualitative variable with a quantitative variable (two means), the Student’s T-test will be used. For comparisons involving more than two means, analysis of variance (ANOVA) will be conducted. When comparing two quantitative variables, the measure of association used will be Pearson’s linear correlation coefficient, and the statistical test will be the Student’s T-test.

Non-parametric tests will be used if the dependent variable does not follow a normal distribution, tailored to the types of variables being compared.

Statistical significance will be set at *p* < 0.05 and the 95% confidence limits for the differences in proportions (qualitative variable) and mean differences (quantitative variable) between the two groups will be calculated.

### Multivariate analysis

To minimize confounding bias in comparisons and to adjust and evaluate possible interactions, a multivariate analysis will be conducted. The most methodologically reasonable model based on the results will be selected and all necessary assumptions will be validated. Statistical significance will be defined as a *p*-value <0.05.

## Data collection of study variables

Primary data collection (study variables) will be conducted through clinical interviews, review of computerized medical records, and complementary tests. Patients diagnosed with SLE, will be conducted by reviewing computerized medical records for the patients diagnosed with “Systemic Lupus Erythematosus” according to the 10th edition of the International Classification of Diseases (ICD-10), who are under follow-up by the Rheumatology Department of the Health Department of Elda.

For the selection of participants in group 1 (SLE), patients will be recruited consecutively from those attending follow-up appointments in the Rheumatology Department. Information from their medical records regarding the inclusion and exclusion criteria will also be considered to avoid including those who are not suitable candidates for the study. Potential candidates will be contacted by phone, informed about the study and invited to participate. Interested individuals will be scheduled for ophthalmology consultations where they will receive all necessary information (information sheet and informed consent) and initiate variable collection.

For the generation of group 2 (healthy controls), patients attending ophthalmology consultations for occupational check-ups, presbyopia evaluations or other minor eye issues not included in the study’s exclusion criteria will be invited to participate. Additionally, companions of patients with SLE attending the consultation will also be invited, provided they meet the inclusion and exclusion criteria. Among these, patients without an SLE diagnosis and without ocular or systemic conditions affecting the CCT, who belong to the Health Department of Elda, will be selected.

Selected patients will be scheduled for ophthalmology consultations in the morning from 9:00 AM to 2:00 PM to avoid diurnal variations in measurements. After providing the information sheet and obtaining informed consent, a clinical interview and the following complementary tests will be conducted in a single visit. An ophthalmological examination will be performed on all subjects by a single observer, including refraction, visual acuity, anterior segment with slit-lamp, fundus examination, pachymetry using Zeiss Cirrus HD-OCT 5000, GAT, Schirmer 2 test, biometry (including keratometry), VF 24–2, and optic nerve OCT pupil dilation with tropicamide. In all cases, corneal OCT will be performed before the instillation of anesthetic and GAT to avoid alterations in the tear film and possible corneal deformations secondary to contact that could interfere with pachymetry. For pachymetry, a minimum of three images will be obtain with the best being selected for analysis. If none of the initial three images meet the quality standards specified by the device, which are indicated by a green signal, additional images will be taken as needed until a sufficiently high-quality image is acquired.

All data regarding the demographic and clinical characteristics of the participants will be recorded in a data collection form, which will serve as a database for statistical analysis using SPSS Statistics. This database will be managed by the principal investigator and will only be accessible to the supervisor, co-supervisor, and the statistical analyst.

## Discussion

### Error control and bias

To minimize random error, a representative sample size was calculated using a consecutive sampling method, as random or systematic sampling is not feasible in this patient population. A multivariate analysis will be conducted to minimize potential confounding bias and interactions.

Furthermore, to enhance the homogeneity, participants from both groups will be selected from the same population sample of the Health Department of Elda and matched based on controllable variables such as sex and age. Patients with conditions or diseases that could independently affect CCT, including keratoconus, corneal edema, diabetes mellitus and COPD, will be excluded from the sample. Regarding keratoconus, individuals with confirmed or suspected diagnosis will be excluded based on the identification of characteristic signs such as Vogt’s striae or Fleischer’s ring during slit-lamp examination, keratometry readings with K readings exceeding 47 diopters and high astigmatism, despite the absence of confirmatory topography.

It is important to note a potential selection bias among the healthy group, as participants are selected based on the absence of a SLE diagnosis without additional confirmation through complementary tests. However, according to the Spanish Society of Rheumatology’s EPISER study, which included 4,900 participants to determine the prevalence of rheumatic diseases in Spain, 12 cases of SLE were identified through a telephone interview screening. Participants meeting positive screening criteria underwent further evaluation in rheumatology clinics for diagnostic confirmation, with 11 cases already diagnosed prior to screening (20). Therefore, given the estimated low prevalence of SLE, the likelihood of misclassifying patients without self-reported or documented SLE in their medical history is low (1/12).

To minimize measurement bias, data for the primary objective will be collected through clinical interviews using validated and calibrated instruments. Additionally, all participants will undergo standardized evaluations conducted by the same investigator, ensuring consistency between groups.

### Limitations

The main study limitation is the lack of prior knowledge regarding the required sample size. Therefore, a pilot study was conducted to determine the minimum sample size necessary for representativeness. Furthermore, selecting participants consecutively, a form of non-probabilistic sampling, may not fully achieve the desired representativeness, thereby potentially limiting the generalizability of the findings.

The study design’s cross-sectional nature presents a limitation in terms of temporality, as both disease and exposure data are collected simultaneously, precluding the establishment of a cause-and-effect relationship due to the absence of temporal sequencing. Additionally, it remains unknown whether CCT varies over the course of the disease or remains stable. Therefore, if a significant difference in CCT between groups and association between CCT and SLE are found upon study completion, a prospective study will be considered.

Finally, a limitation associated with CCT measurement using the Zeiss Cirrus HD-OCT 5000 is that measurements are taken from the tear film to the Descemet membrane (21), which may result in less precise measurements for patients with ocular surface pathology, such as reduced tear production or a decreased tear meniscus. To minimize potential measurement bias, artificial tears will be administered to all patients before performing pachymetry. While OCT is not considered the gold standard for CCT measurement compared to ultrasound pachymetry, both devices are considered interchangeable and quantification of CCT using corneal OCT offers several advantages over USP, including faster results acquisition, reduced need for patient cooperation and the elimination of topical anesthesia requirement, thereby minimizing risks of epithelial damage, corneal deformation and potential ocular contamination (21). Additionally, utilizing OCT for CCT measurement enables the use of a single device for multiple assessments, as OCT is already routinely employed for other evaluations in daily clinical practice. This streamlines the process, enhancing efficiency and reducing the need to transfer the patient between devices. Therefore, opting for OCT aligns with the increasing demand within the scientific community and among patients for faster and less invasive tests, without compromising measurement accuracy.
